# Synthesis, molecular docking and molecular dynamic simulation studies of 2-chloro-5-[(4-chlorophenyl)sulfamoyl]-*N*-(alkyl/aryl)-4-nitrobenzamide derivatives as antidiabetic agents

**DOI:** 10.1186/s13065-020-00703-4

**Published:** 2020-08-09

**Authors:** Samridhi Thakral, Rakesh Narang, Manoj Kumar, Vikramjeet Singh

**Affiliations:** 1 Department of Pharmaceutical Sciences, Guru Jambheshwar University of Science and Technology, Hisar, 125001 India; 2 Institute of Pharmaceutical Sciences, Kurukshetra University, Kurukshetra, 136118 Haryana India

**Keywords:** α-Glucosidase, α-Amylase, Molecular docking, Molecular dynamic simulations, ADMET

## Abstract

A series of 2-chloro-5-[(4-chlorophenyl)sulfamoyl]-*N*-(alkyl/aryl)-4-nitrobenzamide derivatives **(5a**–**5v)** has been synthesized and confirmed by physicochemical(R_f_, melting point) and spectral means (IR, ^1^HNMR, ^13^CNMR). The results of in vitro antidiabetic study against α-glucosidase indicated that compound **5o** bearing 2-CH_3_-5-NO_2_ substituent on phenyl ring was found to be the most active compound against both enzymes. The electron donating (CH_3_) group and electron withdrawing (NO_2_) group on a phenyl ring highly favoured the inhibitory activity against these enzymes. The docking simulations study revealed that these synthesized compounds displayed hydrogen bonding, electrostatic and hydrophobic interactions with active site residues. The structure activity relationship studies of these compounds were also corroborated with the help of molecular modeling studies. Molecular dynamic simulations have been done for top most active compound for validating its α-glucosidase and α-amylase inhibitory potential, RMSD analysis of ligand protein complex suggested the stability of top most active compound **5o** in binding site of target proteins. In silico ADMET results showed that synthesized compounds were found to have negligible toxicity, good solubility and absorption profile as the synthesized compounds fulfilled Lipinski’s rule of 5 and Veber’s rule.
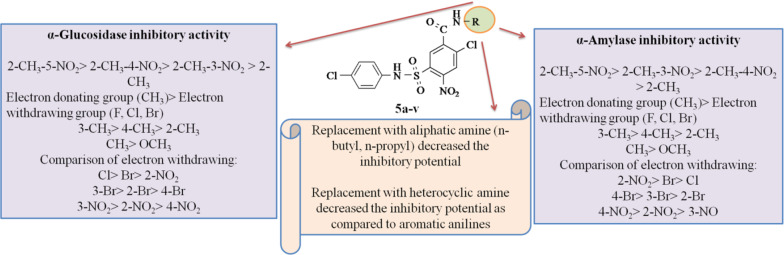

## Introduction

Diabetes mellitus (DM) is a complex metabolic disorder resulting either due to relative or absolute deficiency of pancreatic insulin secretion or insensitivity to insulin action, ensuing in postprandial hyperglycemia and assorted diabetic complications [1, 2]. According to World Health Organization reports, at present around 250 million peoples are living with diabetes and this number is expected to be more than 366 million by 2030 [3] and these statistics are predicted to reach 592 million by 2035 of which 46% may still remain undiagnosed. The reduction of postprandial hyperglycemia by inhibiting carbohydrate hydrolyzing enzymes in gastrointestinal tract is one of the promising approaches for management of diabetes [4, 5]. α-Amylase is involved in hydrolyzing long chain of starch and α-glucosidase release glucose into the small intestine by breaking down oligosaccharides and disaccharides [2, 6]. α-Glucosidase and α-amylase inhibitors reduced postprandial blood glucose level by delaying the hydrolysis of carbohydrate by inhibiting the digestive enzymes [7]. Acarbose, Miglitol and Voglibose are currently available drugs used as α-glucosidase and α-amylase inhibitors, but due to their deleterious side effects such as abdominal distention, diarrhoea and bloating, flatulence [8–10] there is need to explore and synthesize new drug candidates for the management of type-II diabetes mellitus with no or low risk of side effects.

The sulphonamide moiety (–SO_2_NH_2_) is an effective pharmacophore revealing the clinical and medicinal importance of sulphonamide drugs in the field of drug discovery [11]. The lead molecules bearing sulphonamide structure exhibited diverse biological properties viz. antibacterial [12, 13], diuretics, carbonic anhydrase (CA) inhibitors [14], antithyroid, antidiabetic [11, 15, 16], anticancer [17], antitubercular [18], selective Cox II inhibitors [19], anti-inflammatory [20], aldose reductase inhibitor [21], anti-oxidant [22], and anticancer [20] etc. Benzamides are the carbonic acid amide of benzoic acid and have also been described for exhibiting various biological activities i.e. antimicrobial [23, 24], anti-inflammatory [25], anticancer [26, 27], antidiabetic [28], antidepressant, antitubercular [29], anticonvulsant [30] and analgesic [31] etc. 2,4-Dichlorobenzoic acid derivatives have also been reported for their antidiabetic potential exhibiting α-glucosidase and α-amylase inhibitory activity, as described in our previous studies [32, 33]. Singh et al., reported the benzamides as glucokinase activators possessing hypoglycaemic activity [34]. Thiazole-2-yland *N-*pyridin-2-yl benzamides from benzoic acids showed glucokinase activation and possessed good antidiabetic potential in animal rat model [35, 36]. A series of sulfamoyl benzamide derivatives have also been reported by Grewal et al., having glucokinase activation potential for the treatment of type 2 diabetes [37]. In view of the vital importance of benzamides in management of type 2 diabetes, we have synthesized a series of 2-chloro-5-[(4-chlorophenyl)sulfamoyl]-*N*-(alkyl/aryl)-4-nitrobenzamides and evaluated its antidiabetic potential in the current report.

## Results and discussion

### Chemistry

**Scheme 1:**
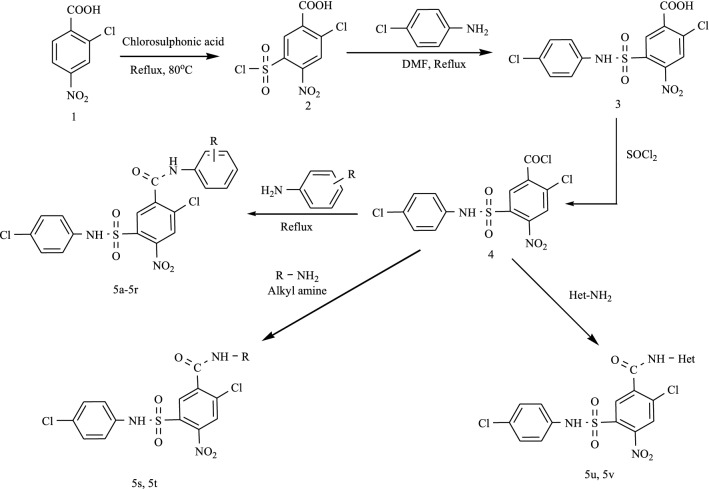
General scheme for synthesis of 2-chloro-5-[(4-chlorophenyl)sulfamoyl]-*N*-(alkyl/aryl)-4-nitrobenzamide derivatives

The 2-chloro-5-(chlorosulfonyl)-4-nitrobenzoic acid (**2**) was prepared from 2-chloro-4-nitro benzoic acid according to our previously reported procedure [32]. The reaction of commercially available *para* chloro substituted aniline with compound **2** in DMF yielded 2-chloro-5-[(4-chlorophenyl)sulfamoyl]-4-nitrobenzoic acid in appropriate amount. The treatment of compound **3** with excess of thionyl chloride in presence of DMF as a catalyst afforded intermediate **4**, which was further refluxed with aromatic/aliphatic/heterocyclic amines in DMF to provide the target compounds **5a**–**5v** (Table 1, Scheme 1).

**Table 1:**
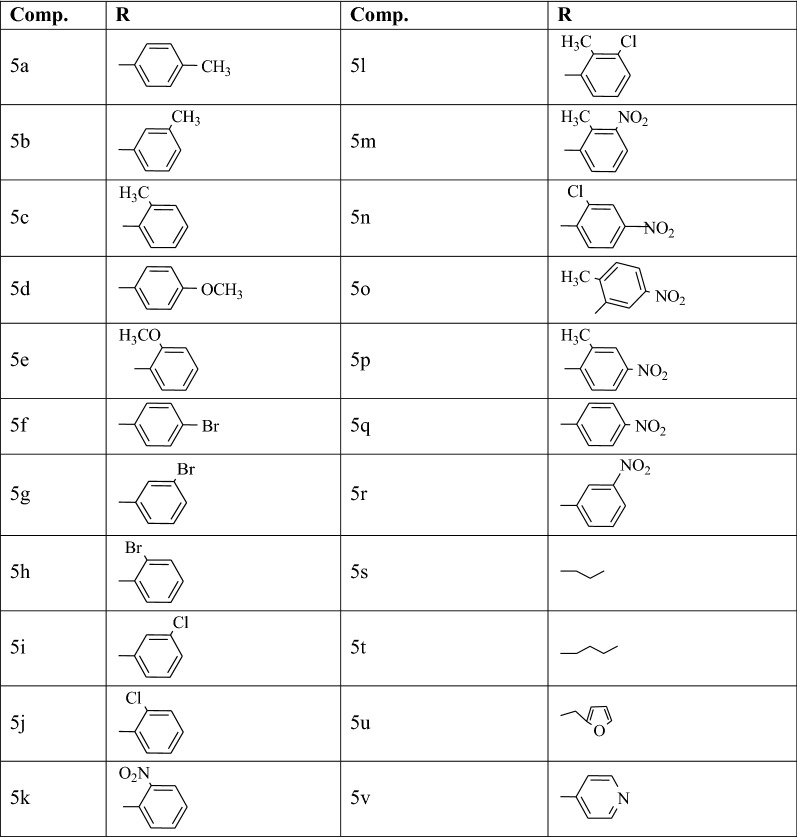
List of synthesized 2-chloro-5-[(4-chlorophenyl)sulfamoyl]-N-(alkyl/aryl)-4-nitrobenzamide compounds

The structure of 2-chloro-5-[(4-chlorophenyl)sulfamoyl]-*N*-(alkyl/aryl)-4-nitrobenzamide compounds was elucidated by IR, ^1^H NMR and ^13^C NMR spectral analysis. The stretching frequency due to NH and carbonyl of amide bond were obtained at 3294–3524 cm^−1^ and 1614–1692 cm^−1^ respectively. The bands around 1302–1398 cm^−1^ and 1127–1183 cm^−1^ were assigned to asymmetric and symmetric stretching of SO_2_ of sulfonamide group respectively. The IR spectrum of synthesized compounds exhibits a band around 1506–1587 cm^−1^ to 1302–1378 cm^−1^ assignable to asymmetric and symmetric stretching of NO_2_. In the ^1^H NMR spectra of compound, singlet for NH protons of SO_2_NH and CONH appeared at δ 3.37–4.08 ppm and δ 10.19–10.81 ppm, respectively. The two aromatic protons of 2-chloro-4-nitro benzoic acid appeared around at δ 8.50 ppm and δ 7.50 ppm. The aromatic protons showed the chemical shift values in region of δ 6.58–8.58 ppm based on their chemical structure. In ^13^C NMR, signals for various carbons appeared in the region of δ 17.72 to 168.51 ppm.

### In vitro antidiabetic evaluation

#### α-Glucosidase inhibitory activity

All the synthesized compounds were tested for their in vitro α-glucosidase inhibitory activity and revealed their varying degree of inhibitory potential with IC_50_ values of 10.75 ± 0.52 to 130.90 ± 2.42 μM (Table 2) as compared to reference acarbose (IC_50_ = 39.48 ± 0.80 μM). The compound **5o** (R = 2-CH_3_-5-NO_2_) was found to be most active among this series of synthesized compounds. Most of the compounds exhibited good inhibitory potential with significant IC_50_ as compared to positive reference.

**Table 2:** α-Glucosidase and α-amylase inhibitory activity (IC_50_) of synthesized derivatives (**5a**–**5v**) and their docking affinity with α-glucosidase (modeled protein) and α-amylase (PDB-1qho)

Comp.	IC_50_ α-glucosidase (µM)	Binding score (α-glucosidase: modeled protein)	IC_50_ α-amylase (µM)	Binding score (α-amylase: 1qho)
5a	31.39 ± 1.66	− 9.4	7.40 ± 0.15	− 8.9
5b	24.78 ± 2.69	− 9.7	5.30 ± 1.23	− 9.0
5c	26.77 ± 1.13	− 9.3	8.00 ± 0.71	− 9.8
5d	38.57 ± 0.01	− 9.2	38.00 ± 0.51	− 9.1
5e	41.75 ± 1.08	− 9.3	50.30 ± 0.21	− 8.7
5f	50.24 ± 0.89	− 9.4	16.00 ± 0.33	− 9.7
5g	35.92 ± 0.60	− 9.6	16.70 ± 0.41	− 9.8
5h	40.64 ± 1.49	− 9.6	19.30 ± 0.63	− 9.7
5i	14.02 ± 0.93	− 9.5	27.12 ± 0.51	− 8.5
5j	15.75 ± 0.90	− 9.6	20.90 ± 1.24	− 9.5
5k	36.93 ± 1.30	− 9.7	12.50 ± 0.91	− 8.8
5l	29.01 ± 0.86	− 9.3	6.30 ± 0.42	− 9.3
5m	24.47 ± 1.23	− 9.4	1.52 ± 0.84	− 9.7
5n	29.54 ± 1.53	− 9.4	35.30 ± 0.45	− 9.6
5o	10.75 ± 0.52	− 9.3	00.90 ± 0.31	− 9.2
5p	19.51 ± 0.43	− 9.4	02.10 ± 0.52	− 9.1
5q	43.88 ± 1.18	− 9.4	11.20 ± 0.67	− 9.0
5r	34.36 ± 0.62	− 9.7	15.30 ± 1.24	− 9.4
5s	106.23 ± 0.61	− 8.2	48.05 ± 0.23	− 7.9
5t	130.90 ± 2.42	− 8.6	55.14 ± 0.71	− 8.3
5u	89.04 ± 1.76	− 8.9	38.20 ± 0.34	− 8.6
5v	52.37 ± 1.92	− 9.0	40.40 ± 0.87	− 8.9
Acarbose	39.48 ± 0.88	− 8.0	5.60 ± 0.30	− 8.4

#### α-Amylase inhibitory activity

All the compounds were also evaluated for α-amylase inhibitory activity and the inhibition potential with IC_50_ values were found in range of 0.90 ± 0.31 μM to 55.14 ± 0.71 μM (Table 2). The compound **5o** showed excellent inhibitory potential against α-amylase with IC_50_ value of 0.90 ± 0.31 μM. Compounds **5b**, **5m**, **5p** showed most significant inhibitory potential against α-amylase with IC_50_ values of 5.30 ± 1.23, 1.52 ± 0.84 and 2.10 ± 0.52 μM, respectively, when compared to acarbose, used as reference compound (IC_50_ = 5.60 ± 0.30 μM).

#### Structure activity relationship

The compound **5o** (R = 2-CH_3_-5-NO_2_) was the most active compound (IC_50_ = 10.75 ± 0.52 μM; 0.90 ± 0.31 μM) which may be due to the presence of electron withdrawing and electron donating group which generate an uniform electron flow, leading the compound to be more active and potent inhibitor against both enzymes. This fact is supported by the similar results of Adegboye et al. [38]. In compounds **5m** (R = 2-CH_3_-3-NO_2_) and **5p** (R = 2-CH_3_-4-NO_2_) difference in inhibitory potential was mainly affected by position of NO_2_ substituent.

However the inhibitory activity increased when the phenyl ring was substituted with CH_3_ at *meta* position, as observed in compound **5b** (IC_50_ = 24.78 ± 2.69 μM; 5.30 ± 1.23 μM) in comparison to compounds **5a** and **5c** having CH_3_ substitution at *para* and *ortho* positions. Further a decrease in inhibitory activity was observed for compounds **5d** (IC_50_ = 38.57 ± 0.01 μM; 38.00 ± 0.51 μM) and **5e** (IC_50_ = 41.75 ± 1.08 μM; 50.30 ± 0.21 μM) bearing OCH_3_ substituted phenyl ring instead of compounds having CH_3_ substituted phenyl ring. The compounds **5f**–**5k**, **5q** and **5r** bearing electron withdrawing groups were found to have considerable inhibitory potential. The results illustrated that compounds **5g** (R = 3-Br), **5i** (R = 3-Cl), **5r** (R = 3-NO_2_), substitution at *meta* position of phenyl ring was found to be most favored for the α-glucosidase inhibitory activity while compounds **5f** and **5q** bearing electron withdrawing groups at *para* position were found to be most favorable for α-amylase inhibitory activity. This fact is supported by Taha et al. [39]. The compounds **5u** (IC_50_ = 89.04 ± 1.76 μM, 38.20 ± 0.34 μM) and **5v** (IC_50_ = 52.37 ± 1.92 μM, 40.40 ± 0.87 μM) substituted with heterocyclic amine displayed reduced inhibitory activities compared to aryl amines. This fact is supported by similar results of Kumar et al. [40] and Charaya et al. [35]. Substituting the compounds with *n*-propyl amine and butyl amine resulted in diminished activity as in compounds **5s** (IC_50_ = 106.23 ± 0.61 μM, 48.05 ± 0.23 μM) and **5t** (IC_50_ = 130.90 ± 2.42 μM, 55.14 ± 0.71 μM). This fact is supported by the similar study on benzamide derivatives by Charaya et al. [35].

### Molecular docking

In silico molecular docking study was performed to investigate binding interactions and to explore binding modes of synthesized compounds with their respective targets. The binding affinities of all the synthesized compounds are reported in Table 2.

### α-Glucosidase enzyme

**Fig. 1:**
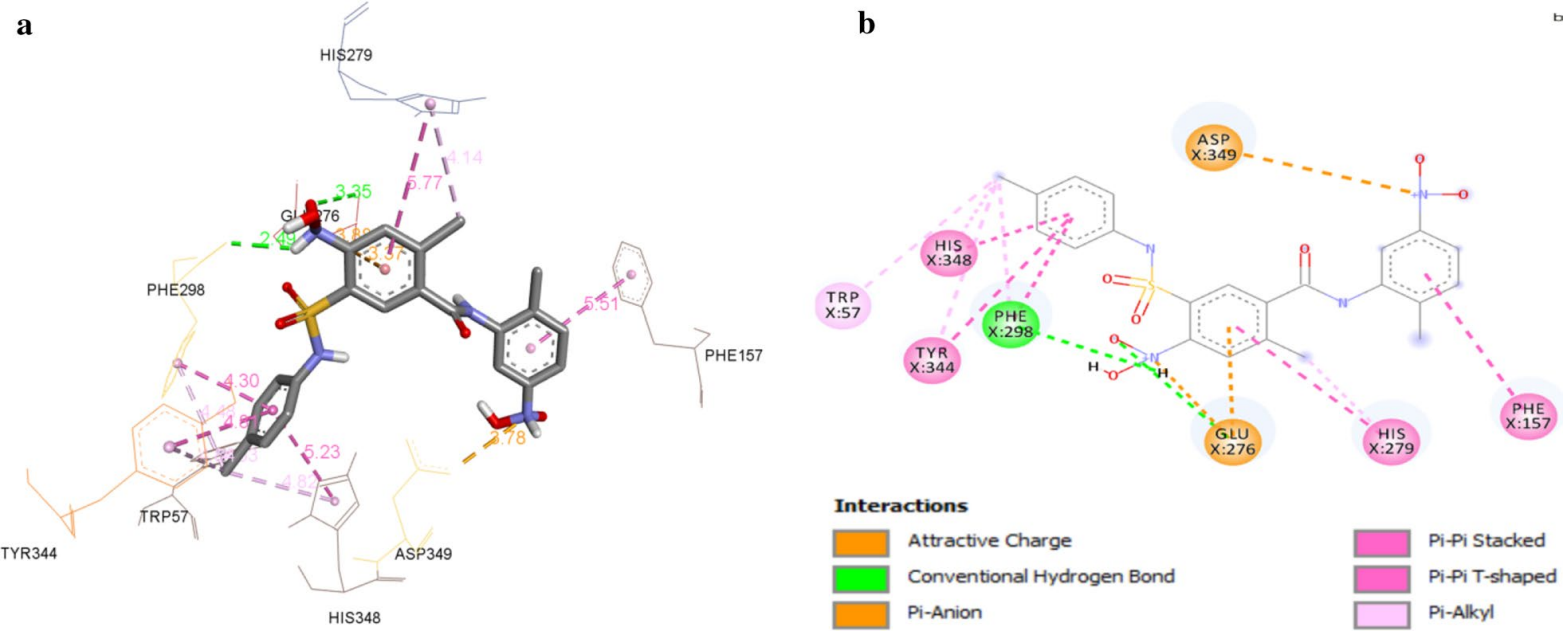
**a** 3D Binding confirmation of compound 5o with active site residues of α-glucosidase. **b** 2D binding confirmation of compound 5o with amino acid residue of nearby active site

The docking results revealed that all the synthesized compounds displayed binding energy ranging from − 9.7 to − 8.0 kcal/mol and depicted various types of significant binding interactions like hydrogen bonding, electrostatic and hydrophobic interactions with the amino acid residues of active site of enzyme. The binding mode of most active compound **5o** and modeled protein is presented in Fig. 1. The oxygen of 2-Cl-4-NO_2_ established hydrogen bonding interaction with Glu:276 amino acid residue at a distance of 3.35 Å whereas Phe:298 amino acid was found to engage in hydrogen bond interactions with both protonated nitrogen of NO_2_ of same with bond lengths of 2.49 Å. The nitrogen of 2-CH_3_-5-NO_2_ substituted compound displayed charge–charge interaction with Asp:349 amino acid residue (3.78 Å) while the nitrogen of 2-Cl-4-NO_2_ presented charge–charge interaction with Glu:276 amino acid residue (3.80 Å). The 2-Cl-4-NO_2_ substituted phenyl ring created pi-anion interaction with residue Glu:276 of modeled protein at a distance of 3.37 Å. It was noticed that Phe:157 residue (5.51 Å) formed pi–pi T shaped interaction with 2-CH_3_-5-NO_2_ substituted phenyl ring and *para* chloro substituted phenyl ring displayed two pi–pi T shaped and one pi–pi stacked interaction with His:348, Tyr:344 and Phe:298 amino acid residues. In addition 2-Cl-4-NO_2_ substituted phenyl ring created pi–pi stacked and pi–pi T shaped interaction with His:279 amino acid residues with bond length of 5.77 Å. Pi-alkyl interactions were established by chlorine of 2-Cl-4-NO_2_ substituted phenyl ring with His:279 residues at a distance of 4.14 Å. The chlorine of *para* chlorosubstituted phenyl ring was found to engage in forming pi–alkyl interactions with Tyr:344, His:348, Phe:298, Trp:57 amino acid residues of modeled protein. The involvement of 2-CH_3_-5-NO_2_ substituted phenyl ring in forming more hydrophobic interactions i.e. pi–pi interactions may be contributing to better activity of compound **5o** as compared to compounds **5n** (R = 2-CH_3_-3-NO_2_) and **5p** (2-CH_3_-4-NO_2_). Comparison of compound **5c** (R = 2-CH_3_) with **5n** (R = 2-CH_3_-3-NO_2_), **5o** (R = 2-CH_3_-5-NO_2_), **5p** (R = 2-CH_3_-4-NO_2_), **5n**, **5o**, **5p** displayed more hydrophobic interactions with Phe:177, Arg:312, Val:108, His:279, Phe:157, His:348, Tyr:344, Phe:298 amino acid residues of modeled protein which may have resulted in their higher inhibitory potential. The binding interaction between compounds **5c** (R = 2-CH_3_) and residues of modeled protein was nearly same as **5a** (R = 4-CH_3_) and **5b** (R = 3-CH_3_). The difference was that *ortho* methyl substituted phenyl ring maintained pi–pi stacked, pi–alkyl and pi–pi T interactions (hydrophobic interactions) with Try:344, His:348, Phe:298, Phe:177, Phe:158, Tyr:344 amino acid residue that made **5c** more active than **5a** and **5b**.

The compound **5e** (R = 2-OCH_3_) formed less number of hydrogen bonding, electrostatic and hydrophobic interactions as compared to compound **5d** (R = 4-OCH_3_), resulting in decreased inhibitory potential of compound **5e**. The binding of compound **5i** (R = 3-Cl) facilitated one more pi–alkyl interaction with other hydrogen bonding, hydrophobic and electrostatic interactions same as that of compound **5j** (R = 2-Cl), which may be contributing to better potential of compound **5i**. Considering the moderately active compound **5r** (R = 3-NO_2_), additional hydrophobic interaction such as pi–pi interactions with amino acid residues were observed as compared to compounds **5k** (R = 2-NO_2_) and **5q** (R = 4-NO_2_). In comparison to compounds bearing aromatic anilines, a decrease in inhibitory potential was observed in compounds **5s** (R = *n*-propyl), and **5t** (R = *n*-butyl), due to less pi–pi interactions between the inhibitory compounds and amino acid residues. The binding interactions of compound **5u** (R = C_4_H_3_O-CH_2_ (2-furfuryl)) with residues of modeled protein were nearly same as that of **5v** (R = C_5_H_5_N-(pyridine-2-yl)) but the difference was that 2-furfuryl ring exhibited pi–pi T shaped interaction with Trp:177 residue and four hydrogen bond interaction with Asp:329, Arg:376, His:90, Trp:93 residues of α-glucosidase with other interactions while compound **5v** formed three hydrogen bond interactions, which made **5u** more active than **5v** against α-glucosidase enzyme.

### α-Amylase enzyme

**Fig. 2:**
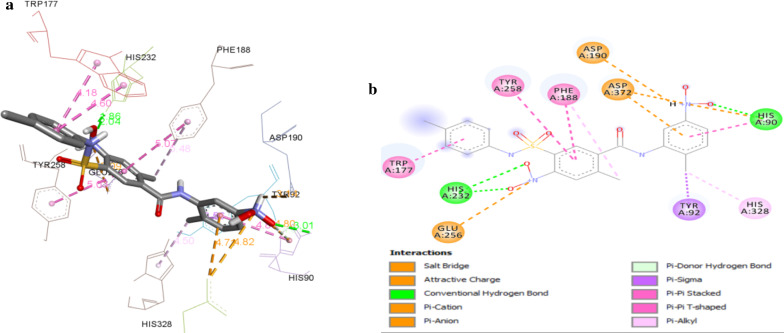
**a** 3D Binding confirmation of compound 5o with active site residues of α-amylase. **b** 2D binding confirmation of compound 5o with amino acid residue of nearby active site

The docking results revealed that all the synthesized compounds displayed binding energy ranging from − 9.8 to − 7.9 kcal/mol. The binding mode of most active compound **5o** and 1qho is presented in Fig. 2. The oxygen of 2-CH_3_-5-NO_2_ established hydrogen bonding interaction with His:90 amino acid residue at a distance of 3.01 Å whereas His:232 amino acid was found to engage in hydrogen bond interactions with both oxygen of NO_2_ of 2-Cl-4-NO_2_ substituted phenyl ring with bond lengths of 2.04 Å and 1.86 Å. The nitrogen of 2-CH_3_-5-NO_2_ displayed charge–charge interaction with Asp:372 amino acid residue (4.82 Å) while the protonated nitrogen of 2-CH_3_-5-NO_2_ presented salt bridge charge–charge interaction with Asp:190 amino acid residue (3.14 Å). The charge–charge interaction was also found between the nitrogen of 2-Cl-4-NO_2_ substituted phenyl ring and Glu:256 amino acid residue with bond length of 5.09 Å. The 2-CH_3_-5-NO_2_ substituted phenyl ring created pi-anion interaction with residue Asp:372 of α-amylase while nitrogen of 2-CH_3_-5-NO_2_ substituted phenyl ring formed pi-cation interaction with His:90 residue. It was shown that His:90 residue (4.88 Å) formed pi–pi T shaped interaction with 2-CH_3_-5-NO_2_ substituted phenyl ring and *para* chloro substituted phenyl ring displayed pi–pi stacked interaction with Trp:177 residue (4.60 Å). In addition 2-Cl-4-NO_2_ substituted phenyl ring created pi–pi stacked and pi–pi T shaped interaction with Tyr:258, Phe:188 amino acid residues with bond lengths of 5.3 Å and 5.07 Å, respectively. The pi–alkyl interactions were established by chlorine of 2-Cl-4-NO_2_ substituted phenyl ring and methyl of 2-CH_3_-5-NO_2_ substituted phenyl ring with Phe:188 and His:328 residues at a distance of 5.48 Å and 4.50 Å, respectively. The CH_3_ of 2-CH_3_-5-NO_2_ substituted phenyl ring was found to engage in forming pi-sigma interaction with Tyr:92 residue (3.56 Å) while oxygen of NO_2_ created pi-donor hydrogen bond with His:90 residue at a distance of 4.01 Å.

The involvement of 2-CH_3_-5-NO_2_ substituted phenyl ring in forming more hydrophobic interactions may be contributing to better activity of compound **5o** as compared to compounds **5n** (R = 2-CH_3_-3-NO_2_) and **5p** (2-CH_3_-4-NO_2_).The comparison of compound **5c** (R =CH_3_) with **5n** (R = 2-CH_3_-3-NO_2_), **5o** (R = 2-CH_3_-5-NO_2_) and **5p** (R = 2-CH_3_-4-NO_2_), **5n**, **5o**, **5p** displayed more electrostatic and hydrophobic interactions with Asp:372, Asp:190, Glu:256, His:90, Trp:177, Tyr:258, Phe:188, His:328 and Tyr:92 residues of α-amylase enzyme, which may have resulted in increase in inhibitory potential. The binding interactions between compound **5b** (R = 3-CH_3_) and residues of α-amylase were nearly same as **5a** (R = 4-CH_3_) and **5c** (R = 2–CH_3_). The difference was that *meta* methyl substituted phenyl ring and methyl group maintained pi–pi stacked, pi-alkyl and pi-sigma interactions (hydrophobic interactions) with Trp:177 amino acid residue which made **5b** more active than **5a** and **5c**. The compound **5e** (R = 2-OCH_3_) formed less number of hydrogen bonding, electrostatic and hydrophobic interactions as compared to compound **5d** (R = 4-OCH_3_), resulting in decrease in inhibitory potential of compound **5e**. The binding of compound **5f** (R = 4-Br) facilitated two pi–pi T shaped and one pi–pi stacked interaction of *para* bromo substituted phenyl ring with Tyr:258, Phe:188, Trp:177 amino acid residues and two pi–pi stacked interactions of *para* chloro substituted phenyl ring with phe:188, Tyr:92 amino acid residues with other interactions, which made compound **5f** more active than compounds **5g** (R = 3-Br) and **5h** (R = 2-Br). Considering the moderate active compound **5q** (R = 4-NO_2_), additional hydrophobic interaction such as pi–pi T shaped, pi–pi stacked interactions with Tyr:258 and Trp:177 residues were observed as compared to compounds **5k** (R = 2-NO_2_) and **5r** (R = 3-NO_2_). In comparison to compounds bearing aromatic anilines, a decrease in inhibitory potential was observed in compounds **5s** (R = *n*-propyl), and **5t** (R = *n*-butyl), due to less pi–pi interactions between the inhibitory compounds and amino acid residues. The binding interaction of compound **5u** (R = C_4_H_3_O-CH_2_ (2-furfuryl)) with residues of α-amylase was nearly same as **5v**(R = C_5_H_5_N- (pyridine-2-yl))but the difference was that 2-furfuryl ring exhibited pi–pi T shaped interaction with Trp:177 residue and four hydrogen bond interactions with Asp:329, Arg:376, His:90, Trp:93 residues of α-amylase with other interactions while compound **5v** formed three hydrogen bond interactions, which made **5u** more active than **5v** against α-amylase.

### Molecular dynamics study

**Fig. 3:**
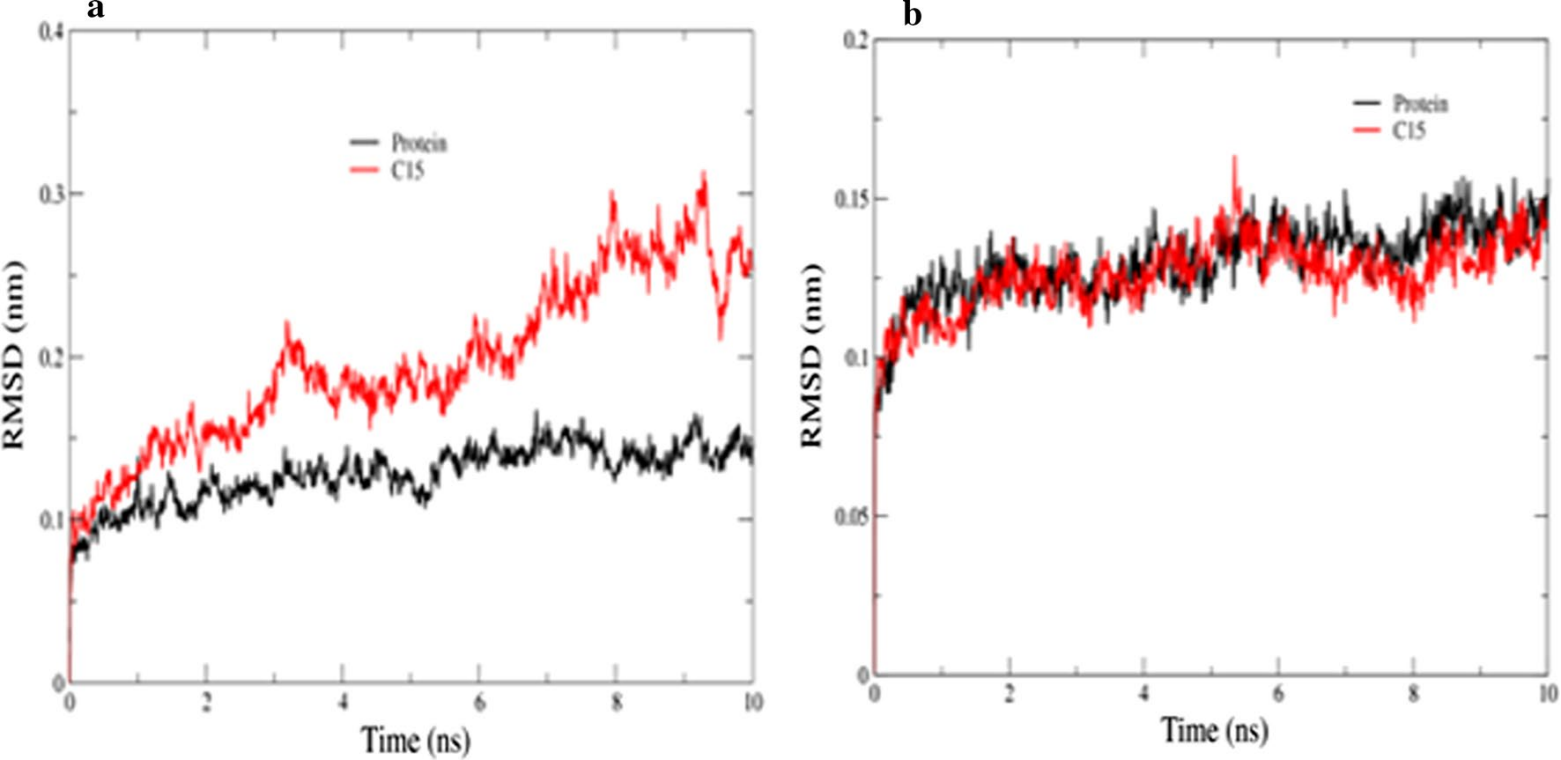
Plot of protein backbone RMSD against time where red color line represent 5o bound modeled protein and black line represent naked protein. **a** For 3aj7 modeled protein with 5o. **b** For 1qho with 5o

**Fig. 4:**
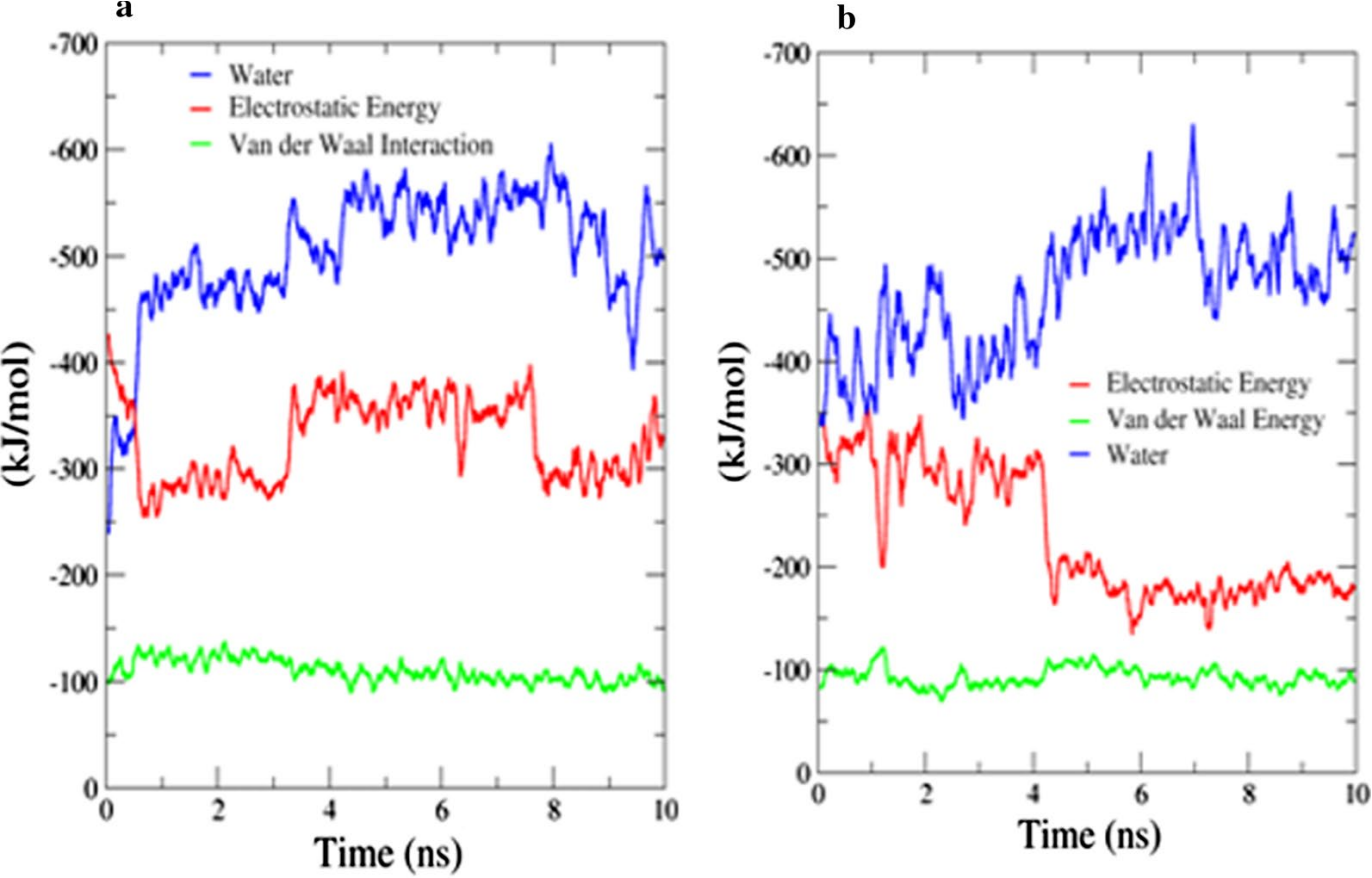
Plot of binding energy (electrostatic and Van der Waal) vs time: **a** For 3aj7 modeled protein with 5o. **b** For 1qho with 5o

A stable protein backbone atoms RMSD vs time is an indication of the near-equilibrium system. As shown in Fig. 3a and b, the protein backbone in both systems attains a constant phase after an initial surge. Whereas, due to the extensive involvement of water molecules (− 500 kJ/mol) the ligand-bound protein backbone has higher RMSD fluctuations compared to the naked protein as represented in Fig. 3a. Figure 4 represent that electrostatic interactions are dominated between the ligand **5o** and protein.

**Fig. 5:**
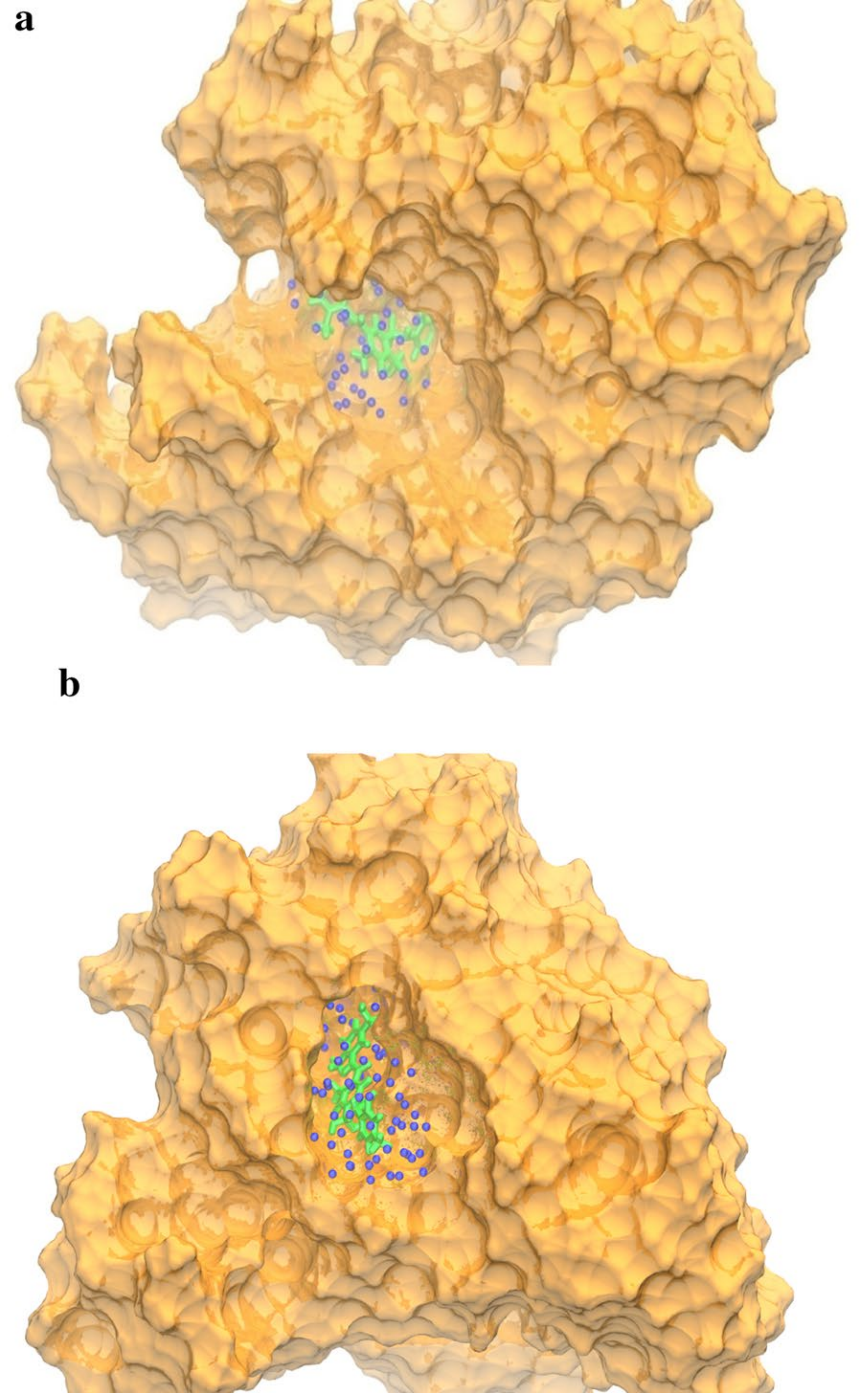
**a** Active site of modeled protein of *S. cerevisiae* α-glucosidase (Golden color) with ligand 5o shown in green color, oxygen atom of water molecule as sphere in blue color. **b** Active site of α-amylase (Golden color) with ligand 5o shown in green color, oxygen atom of water molecule as sphere in blue color

The results obtained from the MD simulations demonstrated that water molecules are predominately involved in ligand–protein interactions (Fig. 5). As shown in the figure, the fall in electrostatic energy that corresponds to the ligand–protein interactions is compensated by the water molecules.

### In silico *ADMET* properties prediction

Lipinski’s rule of five, topological polar surface area, aqueous solubility and number of rotatable bonds, these calculated parameters are presented in Additional file 1: Table S1. The human intestinal absorption values were found in range of 93.10 to 95.93% which established the moderate to good absorption capacity of synthesized compounds and supported their interaction with target cell.

The in vitro Caco-2 cell permeable property in the range of 0.36–0.55 nm/s, in vitro MDCK cell permeability in range of 0.01–0.97 nm/s designated low permeability of target compounds with the concerned cell line. The synthesized compounds displayed values in range of 95.75–100% confirmed their strong binding capacity with proteins. The in vivo blood brain barrier penetration ranges from 0.01 to 0.32 supported their low to moderate distribution in vivo with medium to good penetration capacity (Additional file 1: Table S2). Bioactivity and toxicity risk values of synthesized compounds are illustrated in Additional file 1: Table S3.

## Conclusion

A series of 2-chloro-5-[(4-chlorophenyl)sulfamoyl]-*N*-(alkyl/aryl)-4-nitrobenzamide derivatives (**5a-5v**) has been synthesized and all the compounds were found to possess potent to moderate inhibitory potential against α-glucosidase and α-amylase. Compound **5o** (2-chloro-5-[(4-chlorophenyl) sulfamoyl]-*N*-(2-methyl-5-nitrophenyl)-4-nitrobenzamide) was found to be highly active having fourfold inhibitory potential against α-glucosidase and around six times inhibitory activity against α-amylase in comparison to standard drug acarbose. Molecular docking results of antidiabetic study showed reasonable dock score and binding interactions of synthesized molecules with their respective targets. Analysis of RMSD of ligand protein complex during molecular dynamic simulations suggested stability of the most active compounds **5o** in binding site of respective target proteins i.e. α-glucosidase and α-amylase enzymes. Prediction of computational drug like properties showed that most of synthesized compounds are safe with acceptable ADMET and druggable properties.

## Materials and methods

### Chemicals

The analytical grade chemicals and reagents were used as such in experiments without any purification. Decibel melting point apparatus was used for checking the melting point of the synthesized compounds and are reported as uncorrected. The silica gel-precoated aluminum sheets for thin-layer chromatography (TLC) were employed to keep a vigil of the reaction progress. FT-IR (Diffuse Reflectance Method (DRS) -8000A, Shimadzu, Japan) spectrophotometer was utilized for recording infrared spectra and the Bruker Avance III, 400 MHz NMR spectrometer was employed for nuclear magnetic resonance spectra (^1^H NMR, ^13^C NMR; Chemical shift δ values- ppm). α-Glucosidase from *Saccharomyces cerevisiae* (EC 3.2.1.20, Sigma Aldrich) and α-amylase from malt (232-588-1, HiMedia) have been used for in vitro studies.

### General procedure for synthesis of 2-chloro-5-[(4-chlorophenyl)sulfamoyl]-*N*-(alkyl/aryl)-4-nitrobenzamide (**5a**–**5v**)

#### Synthesis of 2-chloro-5-(chlorosulfonyl)-4-nitro benzoic acid (**2**)

Compound 2 was synthesized from 2-chloro-4-nitro benzoic acid (1) as previously reported method in literature [32].

#### Synthesis of 2-chloro-5-[(4-chlorophenyl)sulfamoyl]-4-nitrobenzoic acid (**3**)

2-Chloro-5-(chlorosulfonyl)-4-nitrobenzoic acid (1 g, 0.003 mol) was refluxed with p-nitro aniline (0.003 mol) using dimethyl formamide as solvent, till the completion of reaction [34]. The reaction progress was monitored by TLC. The reaction mixture was cooled and yielded precipitates were washed and recrystallized.

#### Synthesis of 2-chloro-5-[(4-chlorophenyl)sulfamoyl]-*N*-(alkyl/aryl)-4-nitrobenzamide (**5a**–**5o**)

Compound 3 (0.5 g, 0.0012 mol) was further treated with excess of thionyl chloride in presence of catalytic amount of DMF with calcium chloride (CaCl_2_) guard tube to get 2-chloro-5-[(4-chlorophenyl)sulfamoyl]-4-nitrobenzoylchloride (4). Compound 4 was dissolved in DMF and refluxed with anilines/amines/heterocyclic amines to get the desired products in appropriate yield [41]. After refluxing, mixture was cooled and poured on crushed ice, separated product was filtered and washed with dilute HCl and dried.

### Physicochemical and spectral characterization

#### 2-Chloro-5-[(4-chlorophenyl)sulfamoyl]-*N*-(4-methylphenyl)-4-nitrobenzamide (**5a**)

% Yield: 37.70; m.p.: 90–92 °C; R_f_: 0.81 (Chloroform); FTIR (KBr): ν_max_ (cm^−1^): 3502.79 (N–H str.), 3171.70 (C–H str., Ar), 2977.30, 2889.01 (C–H str., Aliphatic), 1641.45 (C=O), 1600.41 (N–H bend), 1586.48 (asym. NO_2_ str.), 1349.25 (sym. NO_2_ str.), 1315.47 (asym. SO_2_ str.), 1157.31 (sym. SO_2_ str.), 733.44 (C–Cl);^1^HNMR (300 MHz, DMSO-d_6_), δ ppm: 2.15 (s, 3H, CH_3_), 4.01 (s, 1H, NH), 6.78–6.80 (d, 2H, CH of C_3_, C_5_ of –CONH–C_6_H_5_CH_3_–), 7.11–7.13 (d, 2H, CH of C_2_, C_6_ of –CONH–C_6_H_5_CH_3_–), 7.60 (s, 1H, CH of C_6_ of ClNO_2_C_6_H_2_CONH–), 7.88–7.89 (d, 2H, CH of C_2_ and C_6_ of ClC_6_H_4_NH), 8.23 (s, 1H, CH of C_3_ of ClNO_2_C_6_H_2_CONH–), 8.42–8.43 (d, 2H, CH of C_3_ and C_5_ of ClC_6_H_4_NH), 10.69 (s, 1H, NH); ^13^CNMR (300 MHz, DMSO-d_6_), δ ppm: 163.59 (C=O), 148.75 (C–S), 142.92 (C–NO_2_), 136.44 (C–NH), 133.79 (C–Cl), 131.62, 130.79, 130.34, 129.71, 125.76, 123.26, 120.15, 119.55, 117.91, 21.00.

#### 2-Chloro-5-[(4-chlorophenyl)sulfamoyl]-*N*-(3-methylphenyl)-4-nitrobenzamide (**5b**)

% Yield: 50.81; m.p.: 102–104 °C; R_f_: 0.82 (H:E— 8:2); FTIR (KBr): ν_max_ (cm^−1^): 3502.97 (N–H str.), 3117.98 (C–H str., Ar), 2982.94, 2882.00 (C–H str., Aliphatic), 1621.03 (C=O), 1602.20 (N–H bend), 1544.11 (asym. NO_2_ str.), 1370.45 (asym. SO_2_ str.), 1340.55 (sym. NO_2_ str.), 1170.07 (sym. SO_2_ str.), 766.16 (C–Cl); ^1^HNMR (300 MHz, DMSO-d_6_), δ ppm: 2.35 (s, 3H, CH_3_), 3.92 (s, 1H, NH), 5.48 (s, 1H, CH of C_2_ –CONH–C_6_H_5_CH_3_–), 6.88 (s, 1H, CH of C_6_ of ClNO_2_C_6_H_2_CONH–), 7.10–7.12 (d, 2H, CH of C_2_ and C_6_ of ClC_6_H_4_NH), 7.19 (s, 1H, CH of C_3_ of ClNO_2_C_6_H_2_CONH–), 7.23–7.25 (d, 1H, CH of C_6_ of –CONH–C_6_H_5_CH_3_–), 7.35–7.40 (m, 2H, CH of C_4_, C_5_ –CONH–C_6_H_5_CH_3_–), 7.51–7.53 (d, 2H, CH of C_3_ and C_5_ of ClC_6_H_4_NH), 10.31 (s, 1H, NH); ^13^CNMR (300 MHz, DMSO-d_6_), δ ppm: 168.51 (C=O), 151.75 (C–S), 143.40 (C–NO_2_), 139.92 (C–NH), 138.59 (C–Cl), 132.07, 130.56, 130.11, 129.23, 128.68, 124.09, 120.89, 119.21, 115.21, 21.41.

#### 2-Chloro-5-[(4-chlorophenyl)sulfamoyl]-*N*-(2-methylphenyl)-4-nitrobenzamide (**5c**)

% Yield: 84.91; m.p.: 88–90 °C; R_f_: 0.75 (Chloroform); FTIR (KBr): ν_max_ (cm^−1^): 3354.96 (N–H str.), 3197.07 (C–H str., Ar), 2915.46, 2837.25 (C–H str., Aliphatic), 1602.25 (C=O), 1578.76 (N–H bend), 1511.25 (asym. NO_2_ str.), 1374.26 (asym. SO_2_ str.),1348.27 (sym. NO_2_ str.), 1146.44 (sym. SO_2_ str.), 753.00 (C–Cl); ^1^HNMR (300 MHz, DMSO-d_6_), δ ppm: 2.35 (s, 3H, CH_3_), 3.45 (s, 1H, NH), 6.58–6.60 (d, 1H, CH of C_3_ –CONH–C_6_H_5_CH_3_–), 7.26–7.33 (t, 2H, CH of C_4_, C_5_ of –CONH–C_6_H_5_CH_3_–), 7.40–7.41 (d, H, CH of C_6_ –CONH–C_6_H_5_CH_3_–), 7.81 (s, 1H, CH of C_6_ of ClNO_2_C_6_H_2_CONH–), 8.00–8.02 (d, 2H, CH of C_2_ and C_6_ of ClC_6_H_4_NH), 8.36–8.37 (d, 2H, CH of C_3_ and C_5_ of ClC_6_H_4_NH), 8.51 (s, 1H, CH of C_3_ of ClNO_2_C_6_H_2_CONH–), 10.31 (s, 1H, NH); ^13^CNMR (300 MHz, DMSO-d_6_), δ ppm: 162.32 (C=O), 153.39 (C–S), 142.32 (C–NO_2_), 136.24 (C–NH), 133.93 (C–Cl), 131.57, 129.24, 127.88, 125.93, 123.22, 120.56, 117.54, 115.14, 17.72.

#### 2-Chloro-5-[(4-chlorophenyl)sulfamoyl]-*N*-(4-methoxyphenyl)-4-nitrobenzamide (**5d**)

% Yield: 88.69; m.p.: 150–152 °C; R_f_: 0.23 (Chloroform); FTIR (KBr): ν_max_ (cm^−1^): 3438.05 (N–H str.), 3083.26 (C–H str., Ar), 1614.01 (C=O), 1594.19 (N–H bend), 1555.62 (asym. NO_2_ str.), 1352.32 (sym. NO_2_ str.), 1304.87 (asym. SO_2_ str.), 1261.51 (C–O–C str.), 1175.58 (sym. SO_2_ str.), 727.73 (C–Cl);^1^HNMR (300 MHz, DMSO-d_6_), δ: 3.38 (s, 3H, OCH_3_ of –CONH–C_6_H_5_OCH_3_–), 3.89 (s, 1H, NH), 7.02–7.03 (d, 2H, CH of C_3_, C_5_ of –CONH–C_6_H_5_OCH_3_–), 7.34–7.37 (d, 2H, CH of C_2_, C_6_ of –CONH– C_6_H_5_OCH_3_–), 7.52 (s, 1H, CH of C_6_ of ClNO_2_C_6_H_2_CONH–), 7.81–7.83 (d, 2H, CH of C_2_ and C_6_ of ClC_6_H_4_NH), 8.09–8.11 (d, 2H, CH of C_3_ and C_5_ of ClC_6_H_4_NH), 8.50 (s, 1H, CH of C_3_ of ClNO_2_C_6_H_2_CONH–), 10.32 (s, 1H, NH); ^13^CNMR (300 MHz, DMSO-d_6_), δ ppm: 164.63 (C=O), 154.30 (C–S), 144.57 (C–NO_2_), 138.48 (C–NH), 136.24 (C–Cl), 131.58, 129.95, 128.12, 126.09, 124.46, 123.24, 120.20, 32.90.

#### 2-Chloro-5-[(4-chlorophenyl)sulfamoyl]-*N*-(2-methoxyphenyl)-4-nitrobenzamide (**5e**)

% Yield: 85.30; m.p.: 164–166 °C; R_f_: 0.53 (B:EA–7:3); FTIR (KBr): ν_max_ (cm^−1^): 3447.82 (N–H str.), 3012.86 (C–H str., Ar), 1682.45 (C=O), 1597.64 (N–H bend), 1530.54 (asym. NO_2_ str.), 1378.16 (sym. NO_2_ str.), 1307.76 (asym. SO_2_ str.), 1252.68 (C–O–C str.), 1174.67 (sym. SO_2_ str.), 752.65 (C–Cl); ^1^HNMR (300 MHz, DMSO-d_6_), δ ppm: 3.38 (s, 3H, OCH_3_), 3.89 (s, 1H, NH), 7.07–7.09 (d, 1H, CH of C_3_ –CONH–C_6_H_5_OCH_3_–), 7.27.7.33 (m, 3H, CH of C_4_, C_5_, C_6_ of –CONH–C_6_H_5_ OCH_3_–), 7.52 (s, 1H, CH of C_6_ of ClNO_2_C_6_H_2_CONH–), 7.81–7.82 (d, 2H, CH of C_2_ and C_6_ of ClC_6_H_4_NH), 8.28–8.30 (d, 2H, CH of C_3_ and C_5_ of ClC_6_H_4_NH), 8.49 (s, 1H, CH of C_3_ of ClNO_2_C_6_H_2_CONH–), 10.48 (s, 1H, NH); ^13^CNMR (300 MHz, DMSO-d_6_), δ ppm: 164.21 (C=O), 157.32 (C–S), 145.07 (C–NO_2_), 139.07 (C–NH), 136.16 (C–Cl), 131.34, 129.45, 128.24, 127.14, 125.18, 121.68, 121.51, 120.55, 35.60.

#### *N-*(4-Bromophenyl)-2-chloro-5-[(4-chlorophenyl)sulfamoyl]-4-nitrobenzamide (**5f**)

% Yield: 91.30; m.p.: 200–202 °C; R_f_: 0.56 (Chloroform); FTIR (KBr): ν_max_ (cm^−1^): 3392.85 (N–H str.), 3012.55 (C–H str., Ar), 1614.45 (C=O), 1591.36 (N–H bend), 1562.37 (asym. NO_2_ str.), 1353.50 (sym. NO_2_ str.), 1308.72 (asym. SO_2_ str.), 1145.63 (sym. SO_2_ str.), 732.07 (C–Cl), 691.49 (C–Br);^1^HNMR (300 MHz, DMSO-d_6_), δ ppm: 3.60 (s, 1H, NH), 7.46 (s, 1H, CH of C_6_ of ClNO_2_C_6_H_2_CONH–), 7.54–7.55 (d, 2H, CH of C_2_ and C_6_ of ClC_6_H_4_NH), 8.00–8.02 (d, 2H, CH of C_2_, C_6_ of –CONH–C_6_H_5_Br–), 8.24–8.26 (d, 2H, CH of C_3_ and C_5_ of ClC_6_H_4_NH), 8.36–8.37 (d, 2H, CH of C_3_, C_5_ –CONH–C_6_H_5_CH_3_–), 8.51 (s, 1H, CH of C_3_ of ClNO_2_C_6_H_2_CONH–), 10.55 (s, 1H, NH);^13^CNMR (300 MHz, DMSO-d_6_), δ ppm: 166.34 (C=O), 158.33 (C–S), 144.35 (C–NO_2_), 140.21 (C–NH), 135.49 (C–Cl), 132.90, 130.58, 129.89, 129.21, 128.24, 127.08, 120.18.

#### *N-*(3-Bromophenyl)-2-chloro-5-[(4-chlorophenyl)sulfamoyl]-4-nitrobenzamide (**5g**)

% Yield: 44.92; m.p.: 181–183 °C; R_f_: 0.73 (Chloroform); FTIR (KBr): ν_max_ (cm^−1^): 3502.79 (N–H str.), 3058.19 (C–H str., Ar), 1614.45 (C=O), 1588.41 (N–H bend), 1566.61 (asym. NO_2_ str.), 1372.38 (asym. SO_2_ str.), 1302.95 (sym. NO_2_ str.), 1175.63 (sym. SO_2_ str.), 778.76 (C–Cl), 675.80 (C–Br); ^1^HNMR (300 MHz, DMSO-d_6_), δ ppm: 3.71 (s, 1H, NH), 7.37 (s, 1H, CH of C_6_ of ClNO_2_C_6_H_2_CONH–), 7.51 (s, 1H, CH of –CONH–C_6_H_5_Br–), 7.71–7.80 (d, 2H, CH of C_2_ and C_6_ of ClC_6_H_4_NH–), 7.93–7.98 (m, 3H, CH C_4_, C_5_ and C_6_ of –CONH–C_6_H_5_Br–), 8.21–8.23 (d, 2H, CH of C_3_ and C_5_ of ClC_6_H_4_NH), 8.48 (s, 1H, CH of C_3_ of ClNO_2_C_6_H_2_CONH–), 10.40 (s, 1H, NH); ^13^CNMR (300 MHz, DMSO-d_6_), δ ppm: 165.33 (C=O), 157.35 (C–S), 143.24 (C–NO_2_), 139.51 (C–NH), 136.54 (C–Cl), 133.05, 130.77, 129.10, 128.39, 128.17, 127.63, 126.71, 126.12, 121.49.

#### *N-*(2-Bromophenyl)-2-chloro-5-[(4-chlorophenyl)sulfamoyl]-4-nitrobenzamide (**5h**)

% Yield: 55.07; m.p.: 180–182 °C; R_f_: 0.70 (Chloroform); FTIR (KBr): ν_max_ (cm^−1^): 3392.85 (N–H str.), 3028.29 (C–H str., Ar), 1614.62 (C=O), 1594.19 (N–H bend), 1565.96 (asym. NO_2_ str.), 1349.23 (sym. NO_2_ str.), 1377.20 (asym. SO_2_ str.), 1152.15 (sym. SO_2_ str.), 754.34 (C–Cl), 661.15 (C–Br);^1^HNMR (300 MHz, DMSO-d_6_), δ ppm: 3.83 (s, 1H, NH), 6.57–6.61 (t, 1H, CH of C_5_ of –CONH–C_6_H_5_Br–), 6.90–6.92 (d, 1H, CH of C_6_ –CONH–C_6_H_5_Br–), 7.12–7.14 (t, 1H, CH of C_4_ of –CONH–C_6_H_5_Br–), 7.32–7.40 (d, 2H, CH of C_2_ and C_6_ of ClC_6_H_4_NH), 7.51–7.52 (d, 1H, CH of C_3_ of –CONH–C_6_H_5_Br–), 7.82 (s, 1H, CH of C_6_ of ClNO_2_C_6_H_2_CONH–), 8.16–8.18 (d, 2H, CH of C_3_ and C_5_ of ClC_6_H_4_NH), 8.50 (s, 1H, CH of C_3_ of ClNO_2_C_6_H_2_CONH–), 10.39 (s, 1H, NH); ^13^CNMR (300 MHz, DMSO-d_6_), δ ppm: 167.04 (C=O), 156.56 (C–S), 143.06 (C–NO_2_), 138.20 (C–NH), 135.47 (C–Cl), 135.07, 130.23, 129.04, 128.93, 126.13, 125.34, 122.21.

#### 2-Chloro-*N*-(3-chlorophenyl)-5-[(4-chlorophenyl)sulfamoyl]-4-nitrobenzamide (**5i**)

% Yield: 93.25; m.p.: 220–222 °C; R_f_: 0.72 (B:EA– 7:3); FTIR (KBr): ν_max_ (cm^−1^): 3455.53 (N–H str.), 3056.96 (C–H str., Ar), 1687.80 (C=O), 1592.15 (N–H bend), 1542.11 (asym. NO_2_ str.), 1366.59 (sym. NO_2_ str.), 1308.72 (asym. SO_2_ str.), 1165.99 (sym. SO_2_ str.), 782.92 (C–Cl); ^1^HNMR (300 MHz, DMSO-d_6_), δ ppm: 3.39 (s, 1H, NH), 7.51 (s, 1H, CH of C_6_ of ClNO_2_C_6_H_2_CONH–), 7.80–7.83 (d, 2H, CH of C_2_ and C_6_ of ClC_6_H_4_NH), 8.01–8.04 (m, 3H, CH C_4_, C_5_ and C_6_ of –CONH–C_6_H_5_Cl–), 8.23 (s, 1H, CH of –CONH–C_6_H_5_Cl–), 8.30–8.32 (d, 2H, CH of C_3_ and C_5_ of ClC_6_H_4_NH), 8.49 (s, 1H, CH of C_3_ of ClNO_2_C_6_H_2_CONH–), 10.20 (s, 1H, NH); ^13^CNMR (300 MHz, DMSO-d_6_), δ ppm: 167.04 (C=O), 155.28 (C–S), 145.02 (C–NO_2_), 139.44 (C–NH), 136.10 (C–Cl), 132.12, 130.16, 129.12, 128.14, 127.33, 127.17, 126.39, 124.17.

#### 2-Chloro-*N*-(2-chlorophenyl)-5-[(4-chlorophenyl)sulfamoyl]-4-nitrobenzamide (**5j**)

% Yield: 87.71; m.p.: 176–178 °C; R_f_: 0.5 (B:EA– 7:3); FTIR (KBr): ν_max_ (cm^−1^): 3337.42 (N–H str.), 3060.18 (C–H str., Ar), 1665.56 (C=O), 1592.43 (N–H bend), 1533.43 (asym. NO_2_ str.), 1377.20 (sym. NO_2_ str.), 1392.63 (asym. SO_2_ str.), 1173.70 (sym. SO_2_ str.), 755.22 (C–Cl); ^1^HNMR (300 MHz, DMSO-d_6_), δ ppm: 3.89 (s, 1H, NH), 7.44 (s, 1H, CH of C_6_ of ClNO_2_C_6_H_2_CONH–), 7.85–7.90 (m, 3H, CH of C_4_,C_5_ and C_6_ –CONH–C_6_H_5_Cl–), 7.98–8.00 (d, 2H, CH of C_2_ and C_6_ of ClC_6_H_4_NH), 8.21–8.22 (d, 2H, CH of C_3_ and C_5_ of ClC_6_H_4_NH), 8.31–8.33 (d, 1H, CH of C_3_ of –CONH–C_6_H_5_Cl–), 8.52 (s, 1H, CH of C_3_ of ClNO_2_C_6_H_2_CONH–), 10.19 (s, 1H, NH);^13^CNMR (300 MHz, DMSO-d_6_), δ ppm: 166.34 (C=O), 157.02 (C–S), 146.93 (C–NO_2_), 139.24 (C–NH), 135.56 (C–Cl), 131.57, 131.25, 129.53, 128.55, 127.21, 125.24, 121.86.

#### 2-Chloro-5-[(4-chlorophenyl)sulfamoyl]-4-nitro-*N*-(2-nitrophenyl)benzamide (**5k**)

% Yield: 44.61; m.p.: 150–152 °C; R_f_: 0.8 (B:EA– 7:3); FTIR (KBr): ν_max_ (cm^−1^): 3524.01 (N–H str.), 3198.03 (C–H str., Ar), 1614.45 (C=O), 1593.50 (N–H bend), 1563.33 (asym. NO_2_ str.), 1376.23 (asym. SO_2_ str.), 1347.30 (sym. NO_2_ str.), 1146.26 (sym. SO_2_ str.), 746.55 (C–Cl);^1^HNMR (300 MHz, DMSO-d_6_), δ ppm: 3.64 (s, 1H, NH), 7.26–7.28 (d, 1H, CH of C_6_ of –CONH–C_6_H_5_NO_2_–), 7.46–7.48 (d, 1H, CH of C_3_ of –CONH–C_6_H_5_NO_2_–), 7.65 (s, 1H, CH of C_6_ of ClNO_2_C_6_H_2_CONH–), 7.81–7.82 (d, 2H, CH of C_2_ and C_6_ of ClC_6_H_4_NH), 7.90–7.96 (m, 2H, CH of C_4_,C_5_ of –CONH–C_6_H_5_NO_2_–), 8.16–8.19 (d, 2H, CH of C_3_ and C_5_ of ClC_6_H_4_NH), 8.50 (s, 1H, CH of C_3_ of ClNO_2_C_6_H_2_CONH–), 10.30 (s, 1H, NH);^13^CNMR (300 MHz, DMSO-d_6_), δ ppm: 167.03 (C=O), 156.04 (C–S), 149.28 (C–NO_2_), 141.14 (C–NH), 137.43 (C–Cl), 135.29, 133.38, 132.21, 130.67, 130.22, 129.18, 128.15, 126.02.

#### 2-Chloro-*N*-(3-chloro-2-methylphenyl)-5-[(4-chlorophenyl)sulfamoyl]-4-nitrobenzamide (**5l**)

% Yield: 57.62; m.p.: 212–214 °C; R_f_: 0.65 (B:EA– 7:3); FTIR (KBr): ν_max_ (cm^−1^): 3447.82 (N–H str.), 3096.77 (C–H str., Ar), 2947.05, 2885.31 (C–H str., Aliphatic), 1692.29 (C=O), 1592.79 (N–H bend), 1531.51 (asym. NO_2_ str.), 1380.09 (asym. SO_2_ str.), 1306.80 (sym. NO_2_ str.), 1174.67 (sym. SO_2_ str.), 772.81 (C–Cl); ^1^HNMR (300 MHz, DMSO-d_6_), δ ppm: 2.26 (s, H, CH_3_), 3.45 (s, 1H, NH), 7.24–7.30 (m, 2H, CH C_4_, C_5_ andC_6_ of –CONH–C_6_H_5_CH_3_Cl–), 7.63 (s, 1H, CH of C_6_ of ClNO_2_C_6_H_2_CONH–), 7.81–7.82 (d, 2H, CH of C_2_ and C_6_ of ClC_6_H_4_NH), 8.28–8.30 (d, 2H, CH of C_3_ and C_5_ of ClC_6_H_4_NH), 8.58 (s, 1H, CH of C_3_ of ClNO_2_C_6_H_2_CONH–), 10.59 (s, 1H, NH);^13^CNMR (300 MHz, DMSO-d_6_), δ ppm: 165.37 (C=O), 158.36 (C–S), 147.85 (C–NO_2_), 140.10 (C–NH), 138.55 (C–Cl), 137.21, 135.46, 130.80, 129.61, 129.04, 125.15, 121.47, 118.28, 23.57.

#### 2-Chloro-5-[(4-chlorophenyl)sulfamoyl]-*N*-(2-methyl-3-nitrophenyl)-4-nitrobenzamide (**5m**)

% Yield: 38.29; m.p.: 170–172 °C; R_f_: 0.61 (C:T– 9:1); FTIR (KBr): ν_max_ (cm^−1^): 3469.45 (N–H str.), 3095.80 (C–H str., Ar), 2882.55 (C–H str., Aliphatic), 1692.32 (C=O), 1598.62 (N–H bend), 1530.06 (asym. NO_2_ str.), 1351.59 (sym. NO_2_ str.), 1302.76 (asym. SO_2_ str.), 1177.56 (sym. SO_2_ str.), 734.45 (C–Cl);^1^HNMR (300 MHz, DMSO-d_6_), δ ppm: 2.14 (s, H, CH_3_ of –CONH–C_6_H_5_CH_3_NO_2_–), 3.37 (s, 1H, NH), 7.45 (s, 1H, CH of C_6_ of ClNO_2_C_6_H_2_CONH–), 7.94–7.95 (d, 2H, CH of C_2_ and C_6_ of ClC_6_H_4_NH), 8.09–8.13 (m, 2H, CH C_4_, C_5_ andC_6_ of –CONH–C_6_H_5_CH_3_NO_2_–), 8.46–8.47 (d, 2H, CH of C_3_ and C_5_ of ClC_6_H_4_NH), 8.72 (s, 1H, CH of C_3_ of ClNO_2_C_6_H_2_CONH–), 10.50 (s, 1H, NH);^13^CNMR (300 MHz, DMSO-d_6_), δ ppm: 166.48 (C=O), 158.10 (C–S), 146.02 (C–NO_2_), 138.52 (C–NH), 137.46 (C–Cl), 136.78, 136.15, 131.20, 129.89, 129.25, 128.19, 126.71, 126.26, 117.19, 22.76.

#### 2-Chloro-*N*-(2-chloro-4-nitrophenyl)-5-[(4-chlorophenyl)sulfamoyl]-4-nitrobenzamide (**5n**)

% Yield: 79.16; m.p.: 214–216 °C; R_f_: 0.34 (B:EA– 7:3); FTIR (KBr): ν_max_ (cm^−1^): 3461.39 (N–H str.), 3187.49 (C–H str., Ar), 1626.02 (C=O), 1587.77 (asym. NO_2_ str.), 1378.16 (asym. SO_2_ str.), 1320.40 (sym. NO_2_ str.), 1127.06 (sym. SO_2_ str.), 747.47 (C–Cl); ^1^HNMR (300 MHz, DMSO-d_6_), δ ppm: 3.37 (s, 1H, NH), 7.53 (s, 1H, CH of C_6_ of ClNO_2_C_6_H_2_CONH–), 7.80–7.81 (d, 2H, CH of C_2_ and C_6_ of ClC_6_H_4_NH–), 8.11–8.13 (d, 2H, CH of C_3_ and C_5_ of NO_2_C_6_H_4_NH), 8.25–8.26 (d, H, CH of C_6_ of –CONH–C_6_H_5_ClNO_2_), 8.36–8.38 (d, H, CH of C_5_ of –CONH–C_6_H_5_ClNO_2_), 8.51 (s, 1H, CH of C_3_ of ClNO_2_C_6_H_2_CONH–), 8.61 (s, H, CH of C_3_ of –CONH–C_6_H_5_ClNO_2_), 10.53 (s, 1H, NH);^13^CNMR (300 MHz, DMSO-d_6_), δ ppm: 168.41 (C=O), 160.22 (C–S), 145.06 (C–NO_2_), 138.02 (C–NH), 136.61 (C–Cl), 132.42, 130.59, 128.62, 128.41, 126.57, 124.78, 122.38, 118.76, 26.08.

#### 2-Chloro-5-[(4-chlorophenyl)sulfamoyl]-*N*-(2-methyl-5-nitrophenyl)-4-nitrobenzamide (**5o**)

% Yield: 93.61; m.p.: 203–205 °C; R_f_: 0.30 (B:EA– 7:3); FTIR (KBr): ν_max_ (cm^−1^): 3488.67 (N–H str.), 3082.35 (C–H str., Ar), 2979.35, 2899.74 (C–H str., Aliphatic), 1630.01 (C=O), 1511.16 (asym. NO_2_ str.), 1382.02 (asym. SO_2_ str.), 1345.60 (sym. NO_2_ str.), 1138.02 (sym. SO_2_ str.), 737.34 (C–Cl);^1^HNMR (300 MHz, DMSO-d_6_), δ ppm: 2.14 (s, H, CH_3_ of –CONH–C_6_H_5_CH_3_NO_2_–), 3.37 (s, 1H, NH), 7.24–7.29 (m, 2H, CH C_3_, C_4_ of –CONH–C_6_H_5_CH_3_NO_2_–), 7.62 (s, 1H, CH of C_6_ of ClNO_2_C_6_H_2_CONH–), 7.84–7.87 (d, 2H, CH of C_2_ and C_6_ of ClC_6_H_4_NH), 8.32 (d, 2H, CH of C_3_ and C_5_ of ClC_6_H_4_NH), 8.41 (d, H, CH of C_6_, of –CONH–C_6_H_5_CH_3_NO_2_–), 8.29–8.58 (s, 1H, CH of C_3_ of ClNO_2_C_6_H_2_CONH–), 10.67 (s, 1H, NH);^13^CNMR (300 MHz, DMSO-d_6_), δ ppm: 163.88 (C=O), 153.37 (C–S), 147.61(C–NO_2_), 139.52 (C–NH), 137.36 (C–Cl), 130.91, 129.21, 128.35, 127.24, 124.82, 122.63, 118.04, 110.49, 18.53.

#### 2-Chloro-5-[(4-chlorophenyl)sulfamoyl]-*N*-(2-methyl-4-nitrophenyl)-4-nitrobenzamide (**5p**)

% Yield: 56.66; m.p.: 104–106 °C; R_f_: 0.50 (B:EA– 7:3); FTIR (KBr): ν_max_ (cm^−1^): 3473.73 (N–H str.), 3090.98 (C–H str., Ar), 2838.62 (C–H str., Aliphatic), 1640.49 (C=O), 1586.11 (N–H bend), 1529.58 (asym. NO_2_ str.), 1396.49 (asym. SO_2_ str.), 1350.19 (sym. NO_2_ str.), 1154.21 (sym. SO_2_ str.), 740.69 (C–Cl);^1^HNMR (300 MHz, DMSO-d_6_), δ ppm: 2.12 (s, H, CH_3_ of –CONH–C_6_H_5_CH_3_NO_2_–), 3.46 (s, 1H, NH), 7.51 (s, H, CH of C_3_ of –CONH–C_6_H_5_CH_3_NO_2_–), 7.67 (s, 1H, CH of C_6_ of ClNO_2_C_6_H_2_CONH–), 7.84–7.87 (d, 2H, CH of C_2_ and C_6_ of ClC_6_H_4_NH–), 8.21–8.25 (m, 2H, CH C_5_, C_6_ of –CONH–C_6_H_5_CH_3_NO_2_–), 8.47–8.48 (d, 2H, CH of C_3_ and C_5_ of ClC_6_H_4_NH–), 8.64 (s, 1H, CH of C_3_ of ClNO_2_C_6_H_2_CONH–), 10.70 (s, 1H, NH);^13^CNMR (300 MHz, DMSO–d_6_), δ ppm: 167.72 (C=O), 154.62 (C–S), 148.40 (C–NO_2_), 141.67 (C–NH), 135.94 (C–Cl), 130.20, 128.44, 126.61, 124.87, 124.39, 120.86, 112.81, 17.59.

#### 2-Chloro-*N*-(4-chlorophenyl)-5-[(4-chlorophenyl)sulfamoyl]-4-nitrobenzamide (**5q**)

% Yield: 95.24; m.p.: 177–179 °C; R_f_: 0.65 (B:EA— 7:3); FTIR (KBr): ν_max_ (cm^−1^): 3483.02 (N–H str.), 3108.34 (C–H str., Ar), 1633.74 (C=O), 1599.82 (N–H bend), 1530.54 (asym. NO_2_ str.), 1396.49 (asym. SO_2_ str.), 1353.09 (sym. NO_2_ str.), 1183.49 (sym. SO_2_ str.), 754.34 (C–Cl);^1^HNMR (300 MHz, DMSO-d_6_), δ ppm: 3.67 (s, 1H, NH), 7.44–7.46 (d, 2H, CH of C_2_ and C_6_ of –ClC_6_H_4_NH–), 7.60 (s, 1H, CH of C_6_ of ClNO_2_C_6_H_2_CONH–), 7.95–7.96 (d, 2H, CH of C_2_ and C_6_ of –CONHC_6_H_5_Cl–), 8.00–8.02 (d, 2H, CH of C_3_ and C_5_ of –CONH–C_6_H_5_Cl–), 8.24–8.26 (d, 2H, CH of C_3_ and C_5_ of –ClC_6_H_4_NH–), 8.36 (s, 1H, CH of C_3_ of ClNO_2_C_6_H_2_CONH–), 10.63 (s, 1H, NH);^13^CNMR (300 MHz, DMSO-d_6_), δ ppm: 166.17 (C=O), 156.21 (C–S), 149.28 (C–NO_2_), 138.40 (C–NH), 138.06 (C–Cl), 136.11, 131.94, 132.52, 129.89, 126.84, 125.71, 123.20, 112.90.

#### 2-Chloro-5-[(4-chlorophenyl)sulfamoyl]-4-nitro-*N*-(3-nitrophenyl)benzamide (**5r**)

% Yield: 84.37; m.p.: 135-137 °C; R_f_: 0.59 (B:EA- 7:3); FTIR (KBr): ν_max_ (cm^−1^): 3294.47 (N–H str.), 3102.55 (C–H str., Ar), 1618.98 (C=O), 1569.12 (N–H bend), 1549.18 (asym. NO_2_ str.), 1398.42 (asym. SO_2_ str.), 1353.37 (sym. NO_2_ str.), 1133.20 (sym. SO_2_ str.), 732.87 (C–Cl);^1^HNMR (300 MHz, DMSO-d_6_), δ ppm: 4.08 (s, 1H, NH), 7.05 (s, 1H, CH of C_6_ of ClNO_2_C_6_H_2_CONH–), 7.34–7.49 (t, 1H, CH of C_5_ of –CONH–C_6_H_5_NO_2_–), 7.91–7.93 (d, 2H, CH of C_2_ and C_6_ of ClC_6_H_4_NH), 8.00–8.02 (d, 1H, CH of C_6_ of –CONH–C_6_H_5_NO_2_–), 8.24–8.26 (d, 2H, CH of C_3_ and C_5_ of ClC_6_H_4_NH), 8.36–8.39 (d, 1H, CH of C_4_ of –CONH–C_6_H_5_NO_2_–), 8.64 (s, 1H, CH of C_3_ of ClNO_2_C_6_H_2_CONH–), 8.95 (s, 1H, CH of C_2_of –CONH–C_6_H_5_NO_2_–), 10.81 (s, 1H, NH);^13^CNMR (300 MHz, DMSO-d_6_), δ ppm: 165.98 (C=O), 161.31 (C–S), 149.25 (C–NO_2_), 139.61 (C–NH), 132.26 (C–Cl), 130.24, 125.93, 124.56, 123.59, 118.85, 113.87.

#### 2-Chloro-5-[(4-chlorophenyl)sulfamoyl]-4-nitro-*N*-propylbenzamide (**5s**)

% Yield: 57.89; m.p.: 116–118 °C; R_f_: 0.66 (Chloroform); FTIR (KBr): ν_max_ (cm^−1^): 3524.01 (N–H str.), 3089.05 (C–H str., Ar), 2967.82, 2874.42 (C–H str., Aliphatic), 1650.13 (C=O), 1598.78 (N–H bend), 1533.43 (asym. NO_2_ str.), 1372.38 (sym. SO_2_ str.), 1309.34 (sym. NO_2_ str.), 1168.88 (sym. SO_2_ str.), 755.90 (C–Cl). ^1^HNMR (300 MHz, DMSO-d_6_), δ ppm: 1.82–1.90 (m, 7H, –CONH–C_3_H_7_), 3.39 (s, 1H, NH), 7.50 (s, 1H, CH of C_6_ of ClNO_2_C_6_H_2_CONH–), 7.80–7.83 (d, 2H, CH of C_2_ and C_6_ of ClC_6_H_4_NH), 8.21–8.23 (d, 2H, CH of C_3_ and C_5_ of ClC_6_H_4_NH), 8.51 (s, 1H, CH of C_3_ of ClNO_2_C_6_H_2_CONH–), 10.32 (s, 1H, NH); ^13^CNMR (300 MHz, DMSO-d_6_), δ ppm: 162.67 (C=O), 158.75 (C–S), 146.08 (C–NO_2_), 139.51 (C–NH), 135.49 (C–Cl), 132.72, 128.73, 128.06, 126.60, 31.15, 26.04, 21.41.

#### *N*-Butyl-2-chloro-5-[(4-chlorophenyl)sulfamoyl]-4-nitrobenzamide (**5t**)

% Yield: 84.54; m.p.: 111–113 °C; R_f_: 0.54 (Chloroform); FTIR (KBr): ν_max_ (cm^−1^): 3446.85 (N–H str.), 3186.46 (C–H str., Ar), 2959.53, 2871.74 (C–H str., Aliphatic), 1658.64 (C=O), 1597.82 (N–H bend), 1531.51 (asym. NO_2_ str.), 1372.38 (asym. SO_2_ str.), 1309.80 (sym. NO_2_ str.), 1174.67 (sym. SO_2_ str.), 750.37 (C–Cl);^1^HNMR (300 MHz, DMSO-d_6_), δ ppm: 2.08–2.18 (m, 9H, –CONH–C_4_H_9_), 3.71 (s, 1H, NH), 7.46 (s, 1H, CH of C_6_ of ClNO_2_C_6_H_2_CONH–), 7.79–7.80 (d, 2H, CH of C_2_ and C_6_ of ClC_6_H_4_NH), 8.22–8.24 (d, 2H, CH of C_3_ and C_5_ of NO_2_C_6_H_4_NH), 8.50 (s, 1H, CH of C_3_ of ClNO_2_C_6_H_2_CONH–), 10.51 (s, 1H, NH);^13^CNMR (300 MHz, DMSO-d_6_), δ ppm: 160.69 (C=O), 155.52 (C–S), 144.08 (C–NO_2_), 138.84 (C–NH), 136.60 (C–Cl), 133.53, 129.57, 128.48, 127.57, 32.15, 27.34, 18.99.

#### 2-Chloro-5-[(4-chlorophenyl)sulfamoyl]-*N*-[(furan-2-yl)methyl]-4-nitrobenzamide (**5u**)

% Yield: 67.48; m.p.: 191–193 °C; R_f_: 0.40 (B:EA– 7:3); FTIR (KBr): ν_max_ (cm^−1^): 3503.75 (N–H str.), 3056.03 (C–H str., Ar), 2981.34 (C–H str., Aliphatic), 1665.16 (C=O), 1596.15 (N–H bend), 1506.43 (asym. NO_2_ str.), 1396.80 (asym. SO_2_ str.), 1376.23 (sym. NO_2_ str.), 1149.59 (sym. SO_2_ str.), 743.60 (C–Cl);^1^HNMR (300 MHz, DMSO-d_6_), δ: 2.59 (s, 2H, CH of CONH–CH_2_–C_4_H_3_O), 3.71 (s, 1H, NH), 7.61 (s, 1H, CH of C_6_ of ClNO_2_C_6_H_2_CONH–), 7.79–7.87 (m, 3H, CH of C_2_, C_3_ and C4 of CONH–CH_2_–C_4_H_3_O), 7.92–7.93 (d, 2H, CH of C_2_ and C_6_ of ClC_6_H_4_NH), 8.22–8.24 (d, 2H, CH of C_3_ and C_5_ of ClC_6_H_4_NH), 8.50 (s, 1H, CH of C_3_ of ClNO_2_C_6_H_2_CONH–), 10.58 (s, 1H, NH); ^13^CNMR (300 MHz, DMSO–d_6_), δ ppm: 165.97 (C=O), 157.03 (C–S), 145.82 (C–NO_2_), 142.54 (C–NH), 136.72 (C–Cl), 130.46, 129.18, 125.49, 121.85, 120.80, 113.64, 32.59.

#### 2-Chloro-5-[(4-chlorophenyl)sulfamoyl]-4-nitro-*N*-(pyridin-4-yl)benzamide (**5v**)

% Yield: 71.32; m.p.: 197-199 °C; R_f_: 0.31 (B:EA- 7:3); FTIR (KBr): ν_max_ (cm^−1^): 3503.75 (N–H str.), 3113.16 (C–H str., Ar), 1665.56 (C=O), 1598.05 (N–H bend), 1548.50 (asym. NO_2_ str.), 1371.07 (asym. SO_2_ str.), 1316.44 (sym. NO_2_ str.), 1170.81 (sym. SO_2_ str.), 755.14 (C–Cl);^1^HNMR (300 MHz, DMSO-d_6_), δ ppm: 3.71 (s, 1H, NH), 7.57 (s, 1H, CH of C_6_ of ClNO_2_C_6_H_2_CONH-), 7.80–7.83 (d, 2H, CH of C_2_ and C_6_ of ClC_6_H_4_NH–), 7.93–7.96 (d, 2H, CH of C_2_ and C_6_ of –CONH–C_5_H_4_NH–), 8.24–8.26 (d, 2H, CH of C_3_ and C_5_ of ClC_6_H_4_NH–), 8.36–8.39 (d, 2H, C_3_ and C_5_ CH of –CONH–C_5_H_4_NH), 8.50 (s, 1H, CH of C_3_ of ClNO_2_C_6_H_2_CONH–), 10.56 (s, 1H, NH); ^13^CNMR (300 MHz, DMSO–d_6_), δ ppm: 164.70 (C=O), 156.22 (C–S), 148.17 (C–NO_2_), 144.89 (C–NH), 138.96 (C–Cl), 130.34, 128.50, 125.55, 120.17.

### In vitro antidiabetic studies

#### α-Glucosidase inhibitory assay

The method adopted for performing α-glucosidase inhibitory assay was similar to our prevenient study, Thakral and Singh [32]. Graph Pad Prism program, version 5 was employed for calculation of the 50% inhibitory concentration (IC_50_) of all compound [32, 42, 43].

#### α-Amylase inhibitory assay

Xiao et al., and Yoshikawa et al., illustrated a method, with little modification this method has been adopted for measuring the activity [32, 44].

#### Homology modeling

The 3D model for α-glucosidase is developed by comparative homology modeling technique using SWISS-MODEL web server (https://swissmodel.expasy.org/) [45] and then the quality of modeled structure was validated by Ramachandran plot (RAMPAGE) (http://mordred.bioc.cam.ac.uk/~rapper/rampage.php). The details are available in our previous report [32].

### Molecular docking

Ligand molecules were prepared as per reported method [32] using MarvinSketch and AutoDock tools. The crystal structures of α-amylase, 1qho [32, 46] from *Bacillus sterothermophilus*, maltose/acarbose complex downloaded from the protein data bank (http://www.rcsb.org) and α-glucosidase modeled structure [32] was used for docking in antidiabetic evaluation. Docking studies were carried out as reported in our previous study and literature using AutoDock Vina program [32, 47].

### Molecular dynamic simulations

The respective structures placed in the center of the cubic box, the remaining volume of the box was filled by SPCe [48] water molecules. The whole box is then neutralized by adding the respective number of positive and negative ions using GROMACS 5.4 [49] by replacing the equal number of water molecules. Further energy minimization followed by 10 ns equilibration performed by using OPLS [50] force fields integrated into GROMACS 5.4 package to represent the potential energy of the system.

### Computation of drug like parameters and ADMET profiling

Molinspiration (http://www.molinspiration.com/) online tool kit and OSIRIS property explorer was used for computing drug like characteristics from 2D chemical structures of aforementioned compounds [51–54]. Pre-ADMET online server (https://preadmet.bmdrc.kr/) was used for calculating pharmacokinetic parameters like adsorption, distribution, metabolism and excretion and some of the computed properties are human intestinal absorption (HIA %), Caco-2 cell permeability (nm/s), MDCK (Medin-Darbey Canine Kidney Epithelial Cells) cell permeability (nm/s), plasma protein binding (%)¸ blood brain barrier penetration (C. brain/C. blood) and Pgp inhibition [55]. Bioactivity of synthesized compounds was predicted by Molinspiration (http://www.molinspiration.com/) online tool kit [56] and toxicity parameters like mutagenicity, tumorigenicity irritating effects and reproductive effects were computed by OSIRIS property explorer [57].

## Supplementary information

**Additional file 1: Table S1.** Topological polar surface area, aqueous solubility, number of rotatable bonds, and calculated Lipinski’s rule of five for the synthesized 2-chloro-5-[(4-chlorophenyl)sulfamoyl]-*N*-(alkyl/aryl)-4-nitrobenzamide derivatives; **Table S2.** ADME property values of synthesized 2-chloro-5-[(4-chlorophenyl) sulfamoyl]-*N*-(alkyl/aryl)-4-nitrobenzamide derivatives using Pre-ADMET online server; **Table S3.** Bioactivity and toxicity risk of synthesized 2-chloro-5-[(4-chlorophenyl)sulfa-moyl]-*N*-(alkyl/aryl)-4-nitrobenzamide derivatives

## Data Availability

Not applicable.
