# Supplementary material for: Synthesis, molecular docking and molecular dynamic simulation studies of 2-chloro-5-[(4-chlorophenyl)sulfamoyl]-N-(alkyl/aryl)-4-nitrobenzamide derivatives as antidiabetic agents
Source: BMC Chem. 2020 Aug 9;14(1):49. doi: 10.1186/s13065-020-00703-4 (PMC7416410; doi:10.1186/s13065-020-00703-4)
Supplement: Supplementary file 1 — Additional file 1: Table S1. Topological polar surface area, aqueous solubility, number of rotatable bonds, and calculated Lipinski’s rule of five for the synthesized 2-chloro-5-[(4-chlorophenyl)sulfamoyl]-N-(alkyl/aryl)-4-nitrobenzamide derivatives; Table S2. ADME property values of synthesized 2-chloro-5-[(4-chlorophenyl) sulfamoyl]-N-(alkyl/aryl)-4-nitrobenzamide derivatives using Pre-ADMET online server; Table S3. Bioactivity and toxicity risk of synthesized 2-chloro-5-[(4-chlorophenyl)sulfa-moyl]-N-(alkyl/aryl)-4-nitrobenzamide derivatives [file 13065_2020_703_MOESM1_ESM.docx]

**Synthesis, molecular docking and molecular dynamic simulation studies of 2-chloro-5-[(4-chlorophenyl)sulfamoyl]-N-(alkyl/aryl)-4-nitrobenzamide derivatives as antidiabetic agents**

Samridhi Thakral^1^, Rakesh Narang^2^, Manoj Kumar^1^, Vikramjeet Singh^1^*

^1^Department of Pharmaceutical Sciences, Guru Jambheshwar University of Science and Technology, Hisar-125001, India

^2^Institute of Pharmaceutical Sciences, Kurukshetra University, Kurukshetra-136118, Haryana, India

**Additional File: 1**

**Table S1**: Topological polar surface area, aqueous solubility, number of rotatable bonds, and calculated Lipinski’s rule of five for the synthesized 2-chloro-5-[(4-chlorophenyl)sulfamoyl]-N-(alkyl/aryl)-4-nitrobenzamide derivatives

| **Comp.** | **miLog P^a^** | **Log S^b^**  **(mol/L)** | **TPSA^c^**  **(Å^2^)** | **MW^d^** | **nON^e^** | **nOHNH^f^** | **nvoilation^g^** | **nrot^h^** | **Vol** |
| --- | --- | --- | --- | --- | --- | --- | --- | --- | --- |
| 5a | 5.21 | -6.737 | 121.09 | 480.33 | 8 | 2 | 1 | 6 | 369.05 |
| 5b | 5.19 | -6.737 | 121.09 | 480.33 | 8 | 2 | 1 | 6 | 369.05 |
| 5c | 5.17 | -6.737 | 121.09 | 480.33 | 8 | 2 | 1 | 6 | 369.05 |
| 5d | 4.82 | -6.411 | 130.32 | 496.33 | 9 | 2 | 0 | 7 | 378.33 |
| 5e | 4.77 | -6.411 | 130.32 | 496.33 | 9 | 2 | 0 | 7 | 378.33 |
| 5f | 5.57 | -7.227 | 121.09 | 545.20 | 8 | 2 | 2 | 6 | 370.37 |
| 5g | 5.55 | -7.227 | 121.09 | 545.20 | 8 | 2 | 2 | 6 | 370.37 |
| 5h | 5.53 | -7.227 | 121.09 | 545.20 | 8 | 2 | 2 | 6 | 370.37 |
| 5i | 5.42 | -7.129 | 121.09 | 500.75 | 8 | 2 | 2 | 6 | 366.02 |
| 5j | 5.39 | -7.129 | 121.09 | 500.75 | 8 | 2 | 2 | 6 | 366.02 |
| 5k | 4.68 | -6.853 | 166.91 | 511.30 | 11 | 2 | 2 | 7 | 375.82 |
| 5l | 5.79 | -7.473 | 121.09 | 514.77 | 8 | 2 | 2 | 6 | 382.58 |
| 5m | 5.08 | -7.197 | 166.91 | 525.33 | 11 | 2 | 3 | 7 | 392.38 |
| 5n | 5.31 | -7.589 | 166.91 | 545.74 | 11 | 2 | 3 | 7 | 389.36 |
| 5o | 5.08 | -7.197 | 166.91 | 525.33 | 11 | 2 | 2 | 7 | 389.36 |
| 5p | 5.08 | -7.197 | 166.91 | 525.33 | 11 | 2 | 3 | 7 | 389.36 |
| 5q | 4.72 | -6.853 | 166.91 | 511.30 | 11 | 2 | 2 | 7 | 375.82 |
| 5r | 4.70 | -6.853 | 166.91 | 511.30 | 11 | 2 | 2 | 7 | 375.82 |
| 5s | 3.95 | -5.456 | 121.09 | 432.29 | 8 | 2 | 0 | 7 | 331.24 |
| 5t | 4.05 | -5.726 | 121.09 | 446.31 | 8 | 2 | 0 | 8 | 348.04 |
| 5u | 3.72 | -5.891 | 134.23 | 470.29 | 9 | 2 | 0 | 7 | 350.86 |
| 5v | 3.48 | -5.598 | 133.98 | 467.29 | 9 | 2 | 0 | 6 | 348.33 |

^a^Logarithm of partition coefficient between n-octanol and water (miLog P); ^b^Solublity (LogS); ^c^Topological polar surface area (TPSA); ^d^Molecular weight (MW); ^e^Number of hydrogen bond acceptor (nON); ^f^Number of hydrogen bond donor (nOHNH); ^g^Number of violations (nvoilations); ^h^Number of rotatable bonds (nrot).

**Table S2:** ADME property values of synthesized 2-chloro-5-[(4-chlorophenyl)sulfamoyl]-N-(alkyl/aryl)-4-nitrobenzamide derivatives using Pre-ADMET online server.

| **Comp.** | **Human intestinal absorption (HIA, %)** | ***In vitro* Caco-2 cell permeability (nm/s)** | ***In vitro* MDCK cell permeability (nm/s)** | ***In vitro* plasma protein binding (%)** | ***In vivo* blood brain barrier penetration (C.brain/C. blood)** | **Pgp_ inhibition** |
| --- | --- | --- | --- | --- | --- | --- |
| 5a | 95.29 | 0.47 | 0.04 | 100.00 | 0.08 | inhibitor |
| 5b | 95.29 | 0.47 | 0.05 | 100.00 | 0.14 | inhibitor |
| 5c | 95.29 | 0.47 | 0.05 | 100.00 | 0.29 | inhibitor |
| 5d | 95.67 | 0.52 | 0.04 | 100.00 | 0.05 | inhibitor |
| 5e | 95.67 | 0.52 | 0.04 | 100.00 | 0.18 | inhibitor |
| 5f | 95.81 | 0.45 | 0.01 | 97.55 | 0.07 | inhibitor |
| 5g | 95.81 | 0.53 | 0.01 | 98.77 | 0.16 | inhibitor |
| 5h | 95.81 | 0.55 | 0.01 | 95.75 | 0.32 | inhibitor |
| 5i | 95.47 | 0.50 | 0.05 | 100.00 | 0.15 | inhibitor |
| 5j | 95.47 | 0.50 | 0.05 | 100.00 | 0.32 | inhibitor |
| 5k | 93.10 | 0.37 | 0.04 | 99.71 | 0.14 | inhibitor |
| 5l | 95.56 | 0.56 | 0.04 | 100.00 | 0.19 | inhibitor |
| 5m | 93.79 | 0.37 | 0.04 | 100.00 | 0.01 | inhibitor |
| 5n | 95.75 | 0.38 | 0.04 | 100.00 | 0.01 | inhibitor |
| 5o | 93.79 | 0.37 | 0.04 | 100.00 | 0.01 | inhibitor |
| 5p | 93.79 | 0.37 | 0.04 | 100.00 | 0.31 | inhibitor |
| 5q | 95.10 | 0.37 | 0.04 | 100.00 | 0.23 | inhibitor |
| 5r | 93.10 | 0.36 | 0.04 | 100.00 | 0.01 | inhibitor |
| 5s | 95.35 | 0.42 | 0.97 | 100.00 | 0.31 | inhibitor |
| 5t | 95.42 | 0.48 | 0.21 | 100.00 | 0.13 | inhibitor |
| 5u | 95.87 | 0.42 | 0.07 | 100.00 | 0.17 | inhibitor |
| 5v | 95.93 | 0.40 | 0.26 | 100.00 | 0.21 | inhibitor |

**Table S3:** Bioactivity and toxicity risk of synthesized 2-chloro-5-[(4-chlorophenyl)sulfa-moyl]-N-(alkyl/aryl)-4-nitrobenzamide derivatives

| **Comp.** | **GPCR ligand** | **Ion -channel modu-**  **lator** | **Kinase inhibi-tor** | **Nuclear recep-tor ligand** | **Prot-ease inhibi-tor** | **Muta-genic** | **Tumor-igenic** | **Repro-ductive effective** | **Irri-tant** |
| --- | --- | --- | --- | --- | --- | --- | --- | --- | --- |
| 5a | 0.25 | 0.35 | 0.33 | 0.31 | 0.24 | None | None | None | None |
| 5b | 0.26 | 0.39 | 0.33 | 0.31 | 0.25 | None | None | None | None |
| 5c | 0.26 | 0.37 | 0.32 | 0.30 | 0.26 | None | None | None | Low |
| 5d | 0.25 | 0.37 | 0.32 | 0.29 | 0.24 | None | None | None | None |
| 5e | 0.26 | 0.40 | 0.31 | 0.33 | 0.28 | None | None | None | High |
| 5f | 0.30 | 0.38 | 0.33 | 0.38 | 0.29 | None | High | None | None |
| 5g | 0.32 | 0.39 | 0.30 | 0.42 | 0.30 | None | None | None | None |
| 5h | 0.31 | 0.41 | 0.39 | 0.47 | 0.32 | None | None | None | None |
| 5i | 0.22 | 0.32 | 0.29 | 0.29 | 0.20 | None | None | None | High |
| 5j | 0.24 | 0.33 | 0.28 | 0.33 | 0.25 | None | None | None | Low |
| 5k | 0.23 | 0.35 | 0.33 | 0.40 | 0.24 | None | None | None | None |
| 5l | 0.28 | 0.38 | 0.40 | 0.26 | 0.32 | None | None | None | None |
| 5m | 0.27 | 0.39 | 0.39 | 0.23 | 0.30 | None | None | None | None |
| 5n | 0.26 | 0.34 | 0.33 | 0.38 | 0.27 | None | None | None | Low |
| 5o | 0.28 | 0.37 | 0.31 | 0.32 | 0.27 | None | None | None | None |
| 5p | 0.27 | 0.35 | 0.31 | 0.28 | 0.26 | None | High | None | None |
| 5q | 0.23 | 0.31 | 0.30 | 0.28 | 0.20 | None | None | None | None |
| 5r | 0.24 | 0.33 | 0.29 | 0.32 | 0.21 | None | None | None | None |
| 5s | 0.13 | 0.27 | 0.37 | 0.30 | 0.12 | None | None | None | None |
| 5t | 0.12 | 0.25 | 0.36 | 0.28 | 0.10 | None | None | None | None |
| 5u | 0.33 | 0.45 | 0.49 | 0.41 | 0.37 | None | None | None | None |
| 5v | 0.16 | 0.25 | 0.17 | 0.32 | 0.15 | None | None | None | None |
